# *C*-Methylated Spermidine Derivatives: Convenient Syntheses and Antizyme-Related Effects

**DOI:** 10.3390/biom13060916

**Published:** 2023-05-31

**Authors:** Maxim A. Khomutov, Arthur I. Salikhov, Vladimir A. Mitkevich, Vera L. Tunitskaya, Olga A. Smirnova, Sergey P. Korolev, Alexander O. Chizhov, Marina B. Gottikh, Sergey N. Kochetkov, Alex R. Khomutov

**Affiliations:** 1 Engelhardt Institute of Molecular Biology, Russian Academy of Sciences, Vavilov Street 32, Moscow 119991, Russia; makhomutov@mail.ru (M.A.K.); asalihov93@gmail.com (A.I.S.); mitkevich@gmail.com (V.A.M.); ve_tun@mail.ru (V.L.T.); snk1952@gmail.com (S.N.K.); 2 Belozersky Institute of Physico-Chemical Biology, Lomonosov Moscow State University, Leninskie Gory, Moscow 119991, Russia; spkorolev@mail.ru (S.P.K.); mgottikh@gmail.com (M.B.G.); 3 N.D. Zelinsky Institute of Organic Chemistry, Russian Academy of Sciences, Leninskii Prosp. 47, Moscow 119991, Russia; chizhov@ioc.ac.ru

**Keywords:** polyamines, spermidine, spermine, methylated spermidine analogs, synthesis, antizyme dimerization

## Abstract

The biogenic polyamines, spermidine (Spd) and spermine (Spm), are present at millimolar concentrations in all eukaryotic cells, where they participate in the regulation of vitally important cellular functions. Polyamine analogs and derivatives are a traditional and important instrument for the investigation of the cellular functions of polyamines, enzymes of their metabolism, and the regulation of the biosynthesis of antizyme—a key downregulator of polyamine homeostasis. Here, we describe convenient gram-scale syntheses of a set of *C*-methylated analogs of Spd. The biochemical properties of these compounds and the possibility for the regulation of their activity by moving a methyl group along the polyamine backbone and by changing the stereochemistry of the chiral center(s) are discussed.

## 1. Introduction

The biogenic polyamines, spermidine (Spd) and spermine (Spm), are essential and ubiquitous organic polycations that are present in all eukaryotic cells in μM-mM concentrations and are vitally important for the differentiation, proliferation, and normal functioning of cells [1,2]. Disturbances of polyamine metabolism are associated with many diseases [3,4], including malignant tumors, since cancer cells have elevated levels of polyamines [5]. This feature underlies the practical aspect of the biochemistry of polyamines. It is based on fundamental knowledge of the enzymatic mechanisms of Spm and Spd metabolism, the algorithms used to modulate their activity, the intracellular polyamine pool, and the peculiarities of Spm and Spd interaction with the cellular targets.

Spd has the unique function of participating in the post-translational modification of the eIF5A (translation initiation factor), serving as the only donor of the aminobutyl group transferred to the amino group of Lys-50. Subsequent hydroxylation of this aminobutyl fragment leads to the formation of the amino acid hypusine. It was shown that hypusinated eIF5A is essential for the elongation of the proline- and glycine-rich sites of the peptides [6]. Hypusination of eIF5A is a highly conserved process; it is the last to be affected when the cellular polyamine pool decreases, as was demonstrated in a mutant strain of *Saccharomyces cerevisiae*. This explains why Spd is required to support the growth of cells with chronic polyamine deficiency [7]. Trypanothione, which has two glutathione molecules cross-linked by Spd at Gly residues, is another example of a unique Spd derivative. Trypanothione is a vitally important metabolite for many trypanosomatids, such as those causing leishmaniasis, Chagas disease, and African sleeping sickness. Consequently, trypanothione biosynthesis is a promising target for antiprotozoal drug discovery [8].

Such unique transformations and intracellular functions remained unknown for Spm until 2003, when biochemical disorders leading to Snyder–Robinson syndrome were discovered. It was found that the cause of this rare genetic disease is a set of mutations in the SpmSy gene that result in a significant reduction in Spm level and a dramatic increase in Spd content in different organs and tissues [9,10]. It remains unclear if the lack of Spm, the accumulation of Spd, or both are responsible for the negative physiological consequences, such as asthenic builds, intellectual disabilities, osteoporosis, hypotonia, speech abnormalities, and seizures.

Analogs and derivatives of Spm and Spd are important instruments in the investigation of the cellular functions of polyamines; among the most studied are terminally *N*,*N*′-*bis*-alkylated derivatives of Spm. These compounds are SAT1 inducers and, therefore, mediate to deplete the intracellular polyamine pool. Terminally *N*-substituted derivatives of polyamines possess a wide spectrum of activities including antitumor [11], antiparasite [12,13], and antibacterial activity [14,15]. Highly effective inhibitors of the polyamine catabolizing enzymes, SMOX and PAOX ([16,17,18] and Ref. within), and inhibitors of polyamine transport ([19,20,21] and Ref. within) were found among polyamine analogs. Oligoamine analogs that are more effective than Spm and Spd in inducing antizyme (OAZ1) biosynthesis (a small protein, which is a key downregulator of the polyamine pool) were also reported [22,23,24].

Spm and Spd are easily interconvertible. This significantly complicates the studies of their individual cellular functions. *C*-Monomethylated derivatives of Spm and Spd belong to a unique group of functionally active mimetics of polyamines (for reviews, see [25,26]). Their biochemical properties can be controlled by moving the methyl group along the polyamine backbone [27,28,29,30]. Recently, we discovered that a full-length mouse OAZ1 can dimerize in the presence of polyamines and/or Spd *C*-methylated analogs; in the latter case, dimerization efficiency depends on the methyl group position. Moreover, it was shown that the dimerization potential demonstrated by 2-MeSpd was almost negligible [31]. Changing the stereo configuration of the chiral center(s) of *C*-methylated polyamine analogs gives an opportunity to precisely regulate the productivity of their interaction with enzymes and cells. This observation made it possible to discover functionally active metabolically stable Spm and Spd mimetics suitable for studying their individual cellular functions in vitro and in vivo. Recently, we showed that a metabolically stable *R,R*-diastereomer of 1,12-Me_2_Spm represents a promising lead compound in developing a treatment aimed at targeting the molecular mechanisms underlying the pathology of Snyder–Robinson syndrome [32]. *R*-3-MeSpd was a substrate of neither SAT1 nor SpmSy and, therefore, was metabolically stable. However, unlike the *S*-isomer, it was a substrate for the deoxyhypusine synthase (DHS) and, like Spd, supported the growth of cells with chronic polyamine deficiency [33].

**Figure 1:**
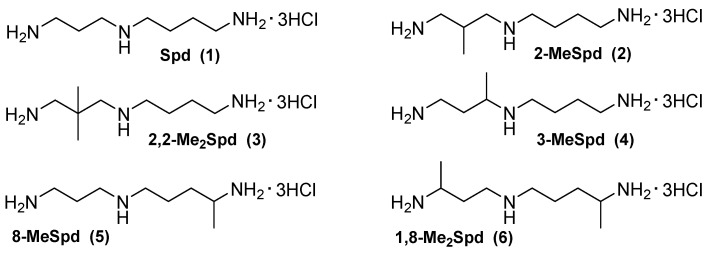
Spd and its *C*-methylated analogs.

It should be noted that the synthesis of racemic *C*-methylated Spd analogs is rather complicated and researchers, as a rule, use independent synthetic routes to obtain each of the analogs (for the review, see [25]). Here, we describe a simple general method for the synthesis of a family of *C*-methylated Spd analogs (Figure 1) with high overall yields, starting from commercially available amino alcohols. We also dwell on the antizyme-related effects of the synthesized compounds.

## 2. Results

### 2.1. Syntheses of 2-MeSpd, 2,2-Me_2_Spd, 3-MeSpd, and 8-MeSpd

**Scheme 1:**
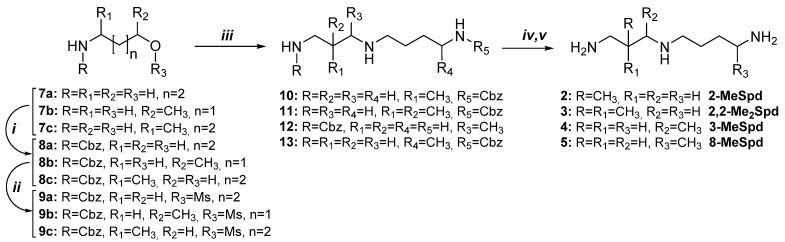
***i***—CbzCl/H_2_O/NaHCO_3_; ***ii***—MsCl/Et_3_N/ CH_2_Cl_2_;***iii***—1. NH_2_CH_2_CH(CH_3_)CH_2_NH_2_/THF for 2-MeSpd; 2. NH_2_CH_2_C(CH_3_)_2_CH_2_NH_2_/THF for 2,2-Me_2_Spd; 3. NH_2_(CH_2_)_3_NH_2_/THF for 8-MeSpd; 4. NH_2_(CH_2_)_4_NH_2_/THF for 3-MeSpd; ***iv***—H_2_/Pd/MeOH/AcOH; ***v***—HCl/EtOH.

Syntheses of *C*-methylated Spd’s **2**–**5** were carried out by a stepwise elongation of the aminomethylene chain. The starting commercially available amino alcohols **7a**–**7c** (Scheme 1) were first converted into *N*-Cbz-amino alcohols **8a**–**8c** with excellent yields under Shotten–Bauman conditions. The obtained **8a**–**8c** were converted to the corresponding mesylates **9a**–**9c** and the latter, without isolation, were treated with an excess of the corresponding diamine in THF to obtain *N*-Cbz-triamines **10**–**13**.

The reaction of alkyl methanesulfonates of primary alcohols **9a** and **9c** with the excess of diamines required 12 h of incubation at 0 °C followed by 16 h at 20 °C; for methanesulfonate of the secondary alcohol **9b**, the reaction was carried out first for 24 h at 20 °C and then for 16 h at 37 °C. All *N*-Cbz-triamines **10**–**13** were isolated by flash column chromatography on SiO_2_ with high yields. Cbz groups were removed by catalytic hydrogenation. The subsequent recrystallization of trihydrochlorides provided target compounds **2**–**5** with overall yields of 40–70%, as calculated from starting amino alcohols **7a**–**7c**, respectively.

### 2.2. Synthesis of 1,8-Me_2_Spd

**Scheme 2:**
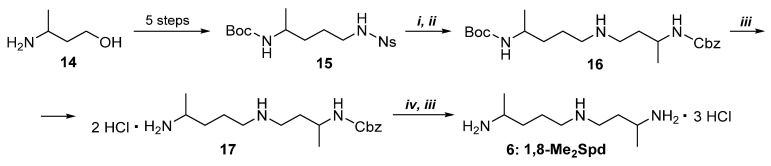
***i***—*N*-(benzyloxycarbonyl)-3-amino-1-bromobutane/DMF/K_2_CO_3_; ***ii***—PhSH/DMF/K_2_CO_3_; ***iii***—HCl/EtOH; ***iv***—H_2_/Pd/AcOH/MeOH. Compound **15** was synthesized according to [29].

Preparation of **6** was performed by the convergent synthetic approach but turned out to be more complicated than the syntheses of **2**–**5** and required a total of 12 steps (Scheme 2). The starting compound was 3-aminobutanol-1 **14**, which was converted into *N*^1^-(2-nitrophenylsulfonyl)-*N*^4^-(*tert*-butyloxycarbonyl)-1,4-diaminopentane **15** in five steps, following previously published protocols [29]. Nosyl derivative **15** was then alkylated with *N*-(benzyloxycarbonyl)-3-amino-1-bromobutane at 20 °C in DMF in the presence of K_2_CO_3_. The nosyl-protecting group was removed one-pot with PhSH, resulting in Boc-Cbz-triamine **16**. Consecutive deprotections of **16** provided target compound **6** in 39% of the overall yield, as calculated from **15**. *N*-(Benzyloxycarbonyl)-3-amino-1-bromobutane was synthesized from 3-aminobutanol-1 **14** in three steps as described [34].

### 2.3. Spermine Binds to mOAZ1 and Induces Its Dimerization In Vitro

Recently, we showed that Spm and Spd bind to full-length mouse recombinant OAZ1 (mOAZ1) carrying His-tag on the *C*-terminus of the polypeptide chain (mOAZ1-6xHis); whereas, under the same conditions, putrescine (1,4-diaminobutane, Put) does not. The stoichiometry of polyamine binding to mOAZ1-6xHis was 2:1, that is, the dimeric (mOAZ1)_2_‒polyamine complex, was formed. The association constant (K_a_) for the complex of Spm with mOAZ1-6xHis was about three times lower than Spd. At the same time, the thermodynamic parameters of complex formation (ΔH and TΔS ratio) for these two polyamines were similar; this suggests that their interaction with mOAZ1 was of the same nature [31]. Surprisingly, among 1-MeSpd, 2-MeSpd, and 3-MeSpd, only 1-MeSpd and 3-MeSpd induced mOAZ1-6xHis dimerization like Spd, whereas 2-MeSpd did not.

Using isothermal titration calorimetry (ITC), we investigated the interaction of Spm with mOAZ1-6xHis, mOAZ1 carrying His-tag on the *N*-terminus of the polypeptide chain (6xHis-mOAZ1), and mOAZ1. 6xHis-mOAZ1 was produced in *E. coli* (Figures S1 and S2) and mOAZ1 was obtained by cleaving 6xHis from 6xHis-mOAZ1 with thrombin (6xHis-mOAZ1 was constructed to have a thrombin-specific site). ITC titration curves and binding isotherms for Spm interacting with mOAZ1-6xHis, 6xHis-mOAZ1, and mOAZ1 are shown in Figure S3. Unexpectedly, 6xHis-OAZ1 did not bind Spm. However, when the *N*-terminal 6xHis-tag was cleaved off with thrombin, the interaction of this protein with Spm was about three times more efficient compared to Spm and OAZ1-6xHis (Table 1).

### 2.4. MeSpds Stabilize the Stem-Loop Region of mOAZ1 mRNA Differently

It was assumed that Spm and Spd may stabilize the hairpin structure of the OAZ1 mRNA, which was located just after the stop-codon and was one of the driving forces of the +1 frameshifting required for the synthesis of full-length OAZ1 [35]. Recently we studied the effects of Spd, 1-MeSpd, 2-MeSpd, and 3-MeSpd on the melting temperature of a model 72-mer 2′-O-Me-oligoribonucleotide (L-OM) containing a +1-frameshifting site, hairpin, and pseudoknot of the mOAZ1 mRNA. It was shown that Spd and its analogs stabilize the oligonucleotide architecture in a concentration-dependent manner. However, 2-MeSpd taken at 200 µM stabilized this hairpin structure less effectively compared with 1-MeSpd, 3-MeSpd, and Spd (Table 2).

Here, we investigated the effects of 8-MeSpd and 2,2-Me_2_Spd on the melting temperature of an L-OM in comparison with Spd and other *C*-methylated Spd analogs. 8-MeSpd stabilized the hairpin structure like Spd. However, sterically hidden 2,2-Me_2_Spd stabilized the hairpin slightly less than Spd and its *C*-methylated analogs, apart from 2-MeSpd (Table 2). This may confirm that the second position of the Spd backbone is one of the “magic sites” responsible for the stabilization of the hairpin structure. This observation is in line with the recently demonstrated inability of 2-MeSpd to form a (mOAZ)_2_-2-MeSpd complex [31].

## 3. Discussion

### 3.1. Chemistry

Analogs and derivatives of Spm and Spd, as a rule, have a fairly simple structure; however, their synthesis, in many cases, turns out to be quite laborious. This is the result of the monotonicity of the target structures and the need to perform the selective transformations of primary and secondary amino groups. In addition, the target compounds are polar, and that makes it difficult to purify them from minor impurities. The key step of the synthesis of Spm, Spd, and their analogs is the building up of the polyamine backbone. Many solution-based and solid-phase methods for the creation of *C*-*N* bonds were adapted to synthesize polyamines, including their *C*-methylated derivatives (for reviews, see [25,36,37,38]).

*gem*-Dimethylated derivatives of Spm and Spd were the first to be synthesized and studied [39]. The investigation of the biochemical potential of 1-MeSpd was a logical development of the work with *gem*-methylated Spd analogs [40]. Originally, 1-MeSpd was obtained by the condensation of *N*-protected 3-aminobutyric acid with Put and the subsequent reduction of the obtained amide. However, because of the poor solubility of the intermediate amide and problems with its reduction and isolation, the target compound 1-MeSpd was obtained only in quantities less than 100 mg, with a total yield of 16% [40]. As an alternative, we proposed a convenient gram-scale 1-MeSpd synthesis by the alkylation of Put in excess with *N*-Cbz-3-amino-1-butyl methanesulfonate and the subsequent deprotection of the amino group that gave a target 1-MeSpd with an overall yield of ~60% (four steps), as calculated from the starting 3-aminobutanol-1 [41].

The first step of the known 2-MeSpd and 3-MeSpd synthesis was Michael’s addition of Put to crotononitrile or methacrylonitrile [29]. Intermediate di-Boc-nitriles were reduced with LiAlH_4_, leading to corresponding di-Boc-triamines, which, after deprotection, gave target 2-MeSpd and 3-MeSpd with >99% purity (HPLC analysis) and overall yields of 34% and 38% (four steps), respectively, as calculated from starting crotononitrile and methacrylonitrile [29]. We also tried to perform a direct reduction of the products of the Michael addition of Put to crotononitrile or methacrylonitrile using Raney nickel. We were not successful with this approach, as the final yields of the pure 2-MeSpd and 3-MeSpd were only around 30% because of the extremely laborious separation of minor impurities from target polyamine analogs [29].

Based on the above synthetic experience, we extended the mesylate approach to the synthesis of 2-MeSpd, 3-MeSpd, 8-MeSpd, and 2,2-Me_2_Spd (Scheme 1). The starting compounds were commercially available amino alcohols, which were converted to *N*-Cbz-methanesulfonates and used to alkylate the corresponding diamine in excess. The subsequent removal of the protecting groups led to target *C*-methylated Spds’ in gram-scale with overall yields of 40 to 70%, as calculated from the corresponding amino alcohols. It should be noted that methanesulfonate **9b** was the least reactive, and slight heating was required to complete the reaction with Put; the yield was less than in the case of primary methanesulfonates, and the formation of minor side products was observed.

The alkylation of *N*-nosylated derivatives of amines was first used for the synthesis of secondary amines back in the 1990s [42]. It was found that in some cases, it turned out to be beneficial compared to the alkylation of primary amines per se. The nosyl group, unlike other sulfamides, is selectively removed under mild conditions by mercaptans, such as thiophenol. We successfully used this approach to obtain symmetrical 1,12-*bis*-methylated analogs of Spm [34], as well as 2,10- and 3,11-*bis*-methylated analogs of Spm [30]. Here, this synthetic approach was extended for the preparation of novel 1,8-Me_2_Spd. This compound complements the panel of *C*-monomethylated analogs of Spd. The alkylation of compound **15** with *N*-(benzyloxycarbonyl)-3-amino-1-bromobutane and the subsequent removal of the protecting groups (Scheme 2) led to target **6** in a 39% overall yield. Although this method involves multiple stages, it is preferable because of the simple isolation and purification of the intermediate and final compounds. Moreover, the removal of the protecting groups at the last step was performed with an almost quantitative yield, which made it possible to obtain the target compound in gram-scale.

Here, a general convenient synthesis of various *C*-methylated analogs of Spd is proposed that makes it possible to obtain corresponding compounds in gram-scale amounts. These Spd analogs are valuable tools for studying the cellular functions of polyamines in vitro and in vivo.

### 3.2. Biochemistry

Homeostasis of polyamines is strictly controlled by differential feedback mechanisms in both positive and negative directions. A small, short-lived protein antizyme (OAZ1) is one of the key downregulators of the intracellular polyamine pool. OAZ1 binds with high affinity to ornithine decarboxylase (ODC), which is the rate-limiting enzyme of polyamine biosynthesis and one of the most short-lived proteins. OAZ1 disrupts active ODC homodimers and targets the ODC subunit for ubiquitin-independent degradation by the 26S proteasome [43]. In addition, OAZ1 regulates both the uptake and excretion of polyamines [44]. Normally, the intracellular amount of OAZ1 is very low, less than 1 ng/mg of the total cytosolic protein in a rat liver [45]. Polyamines are known to increase the efficiency of the translational +1-frameshifting required to pass through the stop codon of OAZ1 mRNA, which is crucial for the synthesis of the full-length protein [46,47].

The nucleotide sequence around the frameshifting site is highly conserved among eukaryotes [48]. Three *cis*-acting elements are known to stimulate mammalian OAZ1 frameshifting: a fifty-nucleotide sequence immediately before the shift site, the stop codon, and the pseudoknot, three nucleotides after the stop codon [46,49]. Deletion analysis and site-directed mutagenesis data indicate that the 5’-element contains several essential regions, each of which contributes to the +1 frameshifting efficiency, and, together, they provide an optimal integral effect [50,51]. However, the molecular mechanism of polyamine-dependent regulation of the frameshifting event is still not completely understood.

Polyamines may stabilize the stem-loop structure of mRNA starting after the stop codon, and these interactions may slow down the elongation speed and allow ribosomes to pass through the stop codon [52]. Recently, using RNA melting temperature analysis, we compared the ability of 1-MeSpd, 2-MeSpd, and 3-MeSpd to stabilize the structure of a model 72-mer 2′-O-Me-oligoribonucleotide containing the +1 frameshifting site, hairpin, and pseudoknot of the mOAZ1 mRNA and demonstrated that 2-MeSpd is a slightly worse stabilizer than Spd, 1-MeSpd, and 3-MeSpd [31]. This correlates with a poor induction of OAZ1 synthesis (Western blot analysis) in DU145 cells grown in the presence of 2-MeSpd [31]. It may be assumed that the second position of the Spd molecule is essential for the stabilization of the hairpin structure of OAZ1 mRNA; indeed, among the studied *C*-methylated Spd analogs, 2,2-Me_2_Spd turned out to be the worst stabilizer, similar to 2-MeSpd (Table 2).

OAZ1 is synthesized and functions in cells with an elevated polyamine pool. However, most studies of OAZ1 were performed in the absence of polyamines. The exception was the study that showed that [^14^C]-Spd binds to yeast OAZ1 and to the conjugate of human OAZ1 with maltose-binding protein [53]. Recently, we demonstrated that Spm, Spd, 1-MeSpd, and 3-MeSpd, but surprisingly not 2-MeSpd, dimerized full-length mOAZ1 that carried a His-tag at the *C*-terminus of the protein (mOAZ1-His_6_) [31]. Here, we studied the interaction of Spm with the full-length mOAZ1 carrying the His-tag at the *N*-terminus of the protein (His_6_-mOAZ1) and demonstrated that such an OAZ1 was not dimerized by Spm. However, when the *N*-terminal His-tag was digested with thrombin, the OAZ1 bound to Spm about three times more efficiently than to the mOAZ1-His_6_, and the dimer (mOAZ1)_2_-Spm complex was formed (Table 1). It may thus be assumed that 20 additional amino acids (MGSSHHHHHHSSGLVPRGSH) at the *N*-terminal region of mOAZ1 may interfere with productive interaction with Spm and prevent the formation of the (mOAZ1)_2_-Spm complex.

*N*-terminally truncated eukaryotic OAZ1s, which are more stable, soluble, and retain the affinity to the ODC subunit, are often used in OAZ1 studies. The structure of rat OAZ1^(87–227)^ was resolved by NMR [54], and the structure of the complex of ODC with human OAZ1^(95–228)^ by X-ray analysis [55]. Functional elements of human OAZ1^(95–228)^ essential for binding the ODC subunit were identified [56]. The *N*-terminal region of OAZ1 is the putative binding site of cyclin D1 [57], but it is unknown whether the *N*-terminal region of OAZ1 plays a role in polyamine sensing, as the polyamine-induced dimerization of *N*-terminally truncated eukaryotic OAZ1 has not been yet studied.

Based on the above, we believe that *C*-methylated analogs of Spm and Spd, especially their chiral derivatives, may be a useful tool for studying the interaction of polyamines with OAZ1 and the investigation of the driving forces of the polyamine-induced +1 frameshifting of mRNA of OAZ1, including the possibility of a stereochemical control of this event.

## 4. Materials and Methods

### 4.1. Materials

*N*^1^-(Benzyloxycarbonyl)-4-amino-butanol-2 **8b** was synthesized as described in [30]; *N*^1^-(2-nitrophenylsulfonyl)-*N*^4^-(*tert*-butyloxycarbonyl)-1,4-diaminopentane **15** was prepared following previously published protocol [29]; *N*-Cbz-4-aminobutanol-1 **8a** was from SigmaAldrich (St. Louis, MO, USA). Methanesulfonyl chloride (Ms-Cl) and benzyl chloroformate (Cbz-Cl) were from Acros (Belgium); 1,4-diaminobutane, 4-aminobutanol-2 and 4-aminopentanol-1 (the latter was freshly distilled before use, bp 111–114 °C/19 Torr) were from Fluka (Switzerland); 1,3-diaminopropane, 2-methyl-1,3-diaminopropane, and 2,2-dimethyl-1,3-diaminopropane were from TCI (Japan). The sequence of mouse OAZ1 was from [58]. The model 72-chain-2′-O-Me-oligonucleotide mOAZ1-PK (5′-UGG UGC UCC UGA UGU CCC UCA CCC ACC CCU GAА GAU CCC AGG UGG GCG AGG GAA CAG UCA GCG GGA UCA CAG-3′) was purchased from DNA-Synthesis LLC (Moscow, Russia). All other reagents, salts, and solvents were of the highest purity and used as supplied by Aldrich and Acros.

Flash chromatography was performed on Kieselgel (40–63 μm, Merck, Germany); elution systems are indicated in the text. TLC was carried out on precoated Kieselgel 60 F_254_ plates (Merck, Germany) using (A) CH_2_Cl_2_-MeOH = 98:2, (B) 1,4-dioxane—25% NH_4_OH = 95:5, (C) *n*-BuOH-AcOH-Py-H_2_O = 4:2:1:2, and (D) dioxane-25% NH_4_OH = 100:1 for elution. ^1^H and ^13^C NMR spectra were measured on a Bruker Avance III (Germany) using tetramethylsilane (TMS) in CDCl_3_ or sodium 3-(trimethylsilyl)-1-propanesulfonate (TSP) in D_2_O as internal standards. Chemical shifts are given in ppm. The letter “*J*” indicates normal ^3^*J*_HH_ couplings, if not specified otherwise, and *J* values are given in Hz. Elemental analysis was performed using a *CHN*-analyser Carlo Erba 1106. High-resolution mass spectra (HR MS) were measured on a Bruker MicrOTOF II instrument using electrospray ionization (ESI) [59]. The measurements were conducted in positive ion mode (interface capillary voltage—4500 V); mass range from *m*/*z* 50 to *m*/*z* 3000; external or internal calibration was conducted with ESI Tuning Mix, Agilent. A syringe injection was used for aqueous solutions of target hydrochlorides or acetonitrile solutions of their protected derivatives (flow rate 3 µL/min). Nitrogen was applied as a dry gas; the interface temperature was set at 180 °C.

### 4.2. Syntheses of 2-MeSpd, 2,2-Me_2_Spd, 3-MeSpd and 8-MeSpd

*N-(Benzyloxycarbonyl)-4-aminopentanol-1* **(8c)**. Benzyl chloroformate (3.5 mL, 25 mmol) was added in five portions at 20 min intervals to a cooled (0 °C) and vigorously stirred mixture of 2.58 g (25 mmol) 4-aminopentanol-1 **7c**, 2 M Na_2_CO_3_ (25 mL), NaHCO_3_ (2.52 g, 25 mmol), and THF (30 mL). Stirring continued for 1 h at 0 °C and for 4 h at room temperature. The organic layer was separated, and the water layer was extracted with CHCl_3_ (3 × 20 mL). The combined organic extracts were concentrated *in vacuo*. The residue was dissolved in CHCl_3_ (50 mL), washed with 1 M HCl (2 × 20 mL), H_2_O (2 × 10 mL), 1 M NaHCO_3_ (10 mL), H_2_O (10 mL), brine (2 × 15 mL), dried (MgSO_4_), filtered, and evaporated to dryness in vacuo. Crude *N*-(benzyloxycarbonyl)-4-aminopentanol-1 (**8c**) was triturated with an ether/hexane (1:3) mixture (80 mL) and left overnight at +4 °C. The precipitate was filtered and dried in vacuo at 1 Torr to produce (**8c**) (5.21g, 88%) as colorless crystals, mp 55–57 °C. *R_f_* 0.22 (A). ^1^H NMR (CDCl_3_) δ: 7.40–7.28 (m, 5Н, C_6_H_5_); 5.08 (s, 2H, CH_2_C_6_H_5_); 4.69 (bs, 1H, Cbz-NH); 3.84–3.70 (m, 1H, NHCH(CH_3_)); 3.69–3.58 (m, 2H, CH_2_OH); 1.80 (bs, 1H, OH); 1.65–1.45 (m, 4H, CHCH_2_CH_2_); 1.15 (d, 3H, *J* = 6.5 Hz, CH_3_). ^13^C NMR (CDCl_3_) δ: 156.31; 136.96; 128.84; 128.41 (2 C); 66.90; 62.86; 47.25; 33.91; 29.25; 21.60. HRESIMS: calculated for C_13_H_20_NO_3_ [M + H]^+^: *m/z* 238.1438. Found: *m/z* 238.1438.

#### 4.2.1. General Method for Synthesis of *N-*Cbz-Protected *C-*Methylated Analogs of Spd (**10**–**13**)

Methanesulfonyl chloride (2.32 mL, 30 mmol) in dry THF (15 mL) was added dropwise within 20 min to a stirred and cooled (0 °C) solution of **8a**–**8c** (30 mmol) and Et_3_N (5.57 mL, 40 mmol) in dry THF (75 mL). Stirring was continued for 1 h at 0 °C, then for 2 h at 20 °C, and the precipitate was filtered off. The filtrate was concentrated in vacuo and the residue was co-evaporated in vacuo with dry toluene (2 × 30 mL) to give **9a**–**9c**.

The cooled (0 °C) solution of diamine (0.3 mol) in dry THF (30 mL) was added to a cooled (0 °C) solution of **9a**–**9c** in dry THF (30 mL). The reaction mixture was kept for 12 h at 4 °C and 24 h at 20 °C (in the case of **9b**, the reaction mixture was additionally kept 12 h at 37 °C). The precipitate was filtered off and the filtrate was concentrated in vacuo. The residue was mixed with 2 M NaOH (30 mL) and the separated oil was extracted with CH_2_Cl_2_ (3 × 30 mL). Combined organic extracts were washed with H_2_O (15 mL), brine (2 × 15 mL), and dried (K_2_CO_3_). Filtrate was evaporated to dryness in vacuo and the residue was purified on a SiO_2_ column using 1,4-dioxane-25% NH_4_OH (elution systems for each of the compounds are in the text). The appropriate fractions were concentrated in vacuo and then dried in vacuo over P_2_O_5_ to give compounds **10**–**13**.

*N^8^-(benzyloxycarbonyl)-1,8-diamino-2-methyl-4-azaoctane* **(10)**. Synthesis was carried out following the above general protocol starting from **8a** (3.4 g, 15 mmol), Ms-Cl (1.16 mL, 15 mmol), Et_3_N (2.8 mL, 20 mmol), and 1,3-diamino-2-methylpropane (15 mL, 0.15 mol). The SiO_2_ column was eluted first with 1,4-dioxane-25% NH_4_OH = 95:5 and then 1,4-dioxane-25% NH_4_OH = 9:1. This afforded **10** (3.12 g, 71%) as a colorless viscous oil, *R_f_* 0.14 (B). ^1^H NMR (CDCl_3_) δ: 7.40–7.27 (m, 5H, C_6_H_5_); 5.63 (bs, 1H, NH-Cbz); 5.05 (s, 2H, CH_2_C_6_H_5_); 3.21–3.10 (m, 2Н, Cbz-NHCH_2_); 2.69–2.38 (m, 6H, CH_2_NHCH_2_ + CH_2_NH_2_); 1.69–1.57 (m, 1H, CH(CH_3_)); 1.54–1.42 (m, 4H, CH_2_CH_2_CH_2_CH_2_); 1.34 (bs, 3H, NH + NH_2_); 0.87 (d, 3H, *J* = 6.5 Hz, CH_3_). ^13^C NMR (CDCl_3_) δ: 156.52; 136.81; 128.44; 128.04; 127.97; 66.42; 54.65; 49.73; 47.12; 40.96; 36.39; 27.82; 27.39; 16.37. HRESIMS: calculated for C_16_H_28_N_3_O_2_ [M + H]^+^: *m/z* 294.2176. Found: *m/z* 294.2174.

*N^1^-(Benzyloxycarbonyl)-1,8-diamino-2,2-dimethyl-4-azaoctane* **(11)**. Synthesis was carried out following the above general protocol starting from **8a** (3.4 g, 15 mmol), Ms-Cl (1.16 mL, 15 mmol), Et_3_N (2.8 mL, 20 mmol), and 2,2-dimethyl-1,3-propanediamine (18 mL, 0.15 mol). The SiO_2_ column was eluted with 95:5 dioxane-25% NH_4_OH to yield **11** (3.59 g, 78%) as a colorless viscous oil, *R_f_* 0.16 (B). ^1^H NMR (CDCl_3_) δ: 7.36–7.27 (m, 5H, C_6_H_5_); 5.49 (bs, 1H, NH-Cbz); 5.06 (s, 2H, CH_2_C_6_H_5_); 3.22–3.10 (m, 2Н, Cbz-NHCH_2_); 2.56 (t, 2H, *J* = 6.6 Hz, CH_2_CH_2_NH); 2.48 (s, 2H, NHCH_2_C); 2.37 (s, 2H, CH_2_NH_2_); 1.34 (bs, 3H, NH + NH_2_); 1.58–1.46 (m, 4H, CH_2_CH_2_CH_2_CH_2_); 1.44 (bs, 3H, NH + NH_2_); 0.83 (s, 6H, (CH_3_)_2_). ^13^C NMR (CDCl_3_) δ: 156.56; 136.82; 128.51; 128.14; 128.05; 66.52; 58.91; 51.52; 50.38; 41.04; 35.29; 27.79; 27.34; 23.86. HRESIMS: calculated for C_17_H_30_N_3_O_2_ [M + H]^+^: *m/z* 308.2333. Found: *m/z* 308.2331.

*N^1^-(Benzyloxycarbonyl)-1,8-diamino-3-methyl-4-azaoctane* **(12)**. Synthesis was carried out following the above general protocol starting from **8b** (2.9 g, 13 mmol), Ms-Cl (1.00 mL, 13 mmol), Et_3_N (2.4 mL, 17.2 mmol), and putrescine (13.2 mL, 0.13 mol). The SiO_2_ column was eluted first with 1,4-dioxane-25% NH_4_OH = 95:5 and then 1,4-dioxane-25% NH_4_OH = 9:1. This gave **12** (2.41 g, 63%) as a colorless viscous oil, *R_f_* 0.26 (B). ^1^H NMR (CDCl_3_) δ: 7.38–7.27 (m, 5H, C_6_H_5_); 6.14 (bs, 1H, NH-Cbz); 5.07 (s, 2H, CH_2_C_6_H_5_); 3.39–3.14 (m, 2Н, Cbz-NHCH_2_); 2.78–2.56 (m, 4H, NHCH_2_ + CH_2_NH_2_); 2.55–2.43 (m, 1H, CH(CH_3_)NH); 1.64–1.37 (m, 6H, CH_2_CH_2_CH_2_CH_2_ + CH_2_CH); 1.25 (bs, 3H, NH + NH_2_); 1.05 (d, 3H, *J* = 6.5 Hz, CH_3_). ^13^C NMR (CDCl_3_) δ: 156.47; 136.92; 128.44; 127.98; 127.94; 66.35; 52.12; 49.83; 42.04; 38.93; 35.73; 31.56; 27.76; 20.37. HRESIMS: calculated for C_16_H_28_N_3_O_2_, [M + H]^+^: *m/z* 294.2176. Found: *m/z* 294.2175.

*N^8^-(benzyloxycarbonyl)-1,8-diamino-4-azanonane* **(13)**. Synthesis was carried out following the above general protocol starting from **8c** (3.5 g, 14.7 mmol), Ms-Cl (1.14 mL, 14.7 mmol), Et_3_N (2.5 mL, 17.9 mmol), and 1,3-diaminopropane (12.6 mL, 0.15 mol). The SiO_2_ column was eluted first with 1,4-dioxane-25% NH_4_OH = 95:5 and then 1,4-dioxane-25% NH_4_OH = 9:1. This gave **7** (3.11 g, 72%), *R_f_* 0.14 (B). ^1^H NMR (CDCl_3_) δ: 7.36–7.27 (m, 5H, C_6_H_5_); 5.24 (bs, 1H, NH-Cbz); 5.04 (s, 2H, CH_2_C_6_H_5_); 3.76–3.58 (m, 1Н, NHCH(CH_3_)); 2.69 (t, 2H, *J* = 6.8 Hz, CH_2_NH_2_); 2.64–2.48 (m, 4H, CH_2_NHCH_2_); 1.64–1.51 (m, 2H, CH_2_CH_2_NH_2_); 1.50–1.36 (m, 4H, CHCH_2_CH_2_); 1.18 (bs, 3H, NH + NH_2_); 1.11 (d, 3H, *J* =6.5 Hz, CH_3_). ^13^C NMR (CDCl_3_) δ: 155.88; 136.76; 128.44; 127.97 (2 C); 66.34; 49.78; 47.80; 46.93; 40.49; 34.76; 33.80; 26.34; 21.12. HRESIMS: Calculated for C_16_H_28_N_3_O_2_, [M + H]^+^: *m/z* 294.2176. Found: *m/z* 294.2176.

#### 4.2.2. General Method for the Syntheses of *C-*Methylated Spermidines Trihydrochloride (**2**–**5**)

A suspension of Pd/black in MeOH (1 mL) was added to a solution of *N*-(benzyloxycarbonyl)-protected analogs of spermidines **10**–**13** (10 mmol) in a mixture of AcOH-MeOH (1:1, 35 mL), and hydrogenation was carried out at atmospheric pressure. The catalyst was filtered off and the filtrate was evaporated to dryness in vacuo. The residue was redissolved in EtOH (20 mL) and diluted with 5 M HCl (10 mL), and the resulting solution was evaporated to dryness in vacuo. The residue was co-evaporated with dry EtOH (2 × 20 mL) and recrystallized from a MeOH-EtOH mixture to give corresponding target compounds **2**–**5**.

*1,8-Diamino-2-methyl-4-azaoctane trihydrochloride (2-MeSpd**)*** **(2)**. Synthesis was carried out following the above general protocol starting from **10** (2.50 g, 8.53 mmol) that gave **8** (2.05 g, 89%) as colorless crystals: mp. 197–199 °C (lit.: 189–192 °C [29]), *R_f_* 0.22 (C). ^1^H NMR (D_2_O) δ: 3.20–3.09 (m, 4H, CHCH_2_NH_2_ + CH_2_NH_2_) 3.08–2.87 (m, 4H, CH_2_NHCH_2_); 2.42–2.24 (m, 1H, CH(CH_3_)); 1.88–1.68 (m, 4H, CH_2_CH_2_CH_2_NH_2_); 1.15 (d, 3H, *J* = 6.8 Hz, CH_3_). ^13^C NMR (D_2_O) δ: 51.28, 48.38, 43.03, 39.47, 30.06, 24.58, 23.26, 14.85. Found, %: C 35.76; Н 9.17; N 15.80. C_8_H_24_Cl_3_N_3_. Calculated, %: C 35.77; Н 9.00; N 15.64. HRESIMS: calculated for C_8_H_22_N_3_ [M + H]^+^: *m/z* 160.1808. Found: *m/z* 160.1811.

*1,8-Diamino-2,2-dimethyl-4-azaoctane trihydrochloride (2,2-Me_2_Spd)* **(3)**. Synthesis was carried out following the above general protocol starting from **11** (2.12 g, 6.9 mmol) that gave **9** (1.77 g, 91%) as colorless crystals: mp. 261–264 °C (lit.: 265 °C [39]), *R_f_* 0.29 (C). ^1^H NMR (D_2_O) δ: 3.19–3.01 (m, 8H, CH_2_CH_2_NH_2_ + NHCH_2_CH_2_ + NH_2_CH_2_C + 2H, CCH_2_NH); 1.90–1.68 (m, 4H, CH_2_CH_2_CH_2_CH_2_); 1.17 (s, 6H, (CH_3_)_2_). ^13^C NMR (D_2_O) δ: 56.00, 49.24, 47.64, 39.46, 33.26, 24.60, 23.00, 22.19. Found, %: C 38.09, H 9.15, N 14.71. C_9_H_26_N_3_Cl_3_. Calculated for, %: C 38.24, H 9.27, N 14.86. HRESIMS: calculated for C_9_H_24_N_3_ [M + H]^+^: *m/z* 174.1965. Found: *m/z* 174.1962.

*1,8-Diamino-3-methyl-4-azaoctane trihydrochloride (3-MeSpd)* **(4)**. Synthesis was carried out following the above general protocol starting from **6** (2.0 g, 6.8 mmol) that gave **10** (1.61 g, 88%) as colorless crystals: mp. 233–235°C (lit.: 231–232 °C [29]), *R_f_* 0.24 (C). ^1^H NMR (D_2_O) δ: 3.50–3.37 (m, 1H, CH(CH_3_)); 3.22–2.99 (m, 6H, H_2_NCH_2_(CH_2_)_2_CH_2_NH + CH_2_NH_2_); 2.27–2.11 (m, 1H, CHCH_2_); 2.03–1.86 (m, 1H, CHCH_2_); 1.83–1.70 (m, 4H, CH_2_CH_2_CH_2_CH_2_); 1.36 (d, 3H, *J* = 6.7 Hz, CH(CH_3_)). ^13^C NMR (D_2_O) δ: 52.69, 44.90, 39.49, 36.56, 30.88, 24.63, 23.62, 15.75. Found, %: C 35.57; Н 9.06; N 15.53. C_8_H_24_Cl_3_N_3_. Calculated, %: C 35.77; Н 9.00; N 15.64. HRESIMS: calculated for C_8_H_22_N_3_ [M + H]^+^: *m/z* 160.1808. Found: *m/z* 160.1806.

*1,8-Diamino-4-azanonane trihydrochloride (8-MeSpd)* **(5)**. Synthesis was carried out following the above general protocol starting from **7** (2.50 g, 8.5 mmol) that gave **11** (2.03 g, 89%) as colorless crystals: mp 198–199°C (lit.: 195–196 °C [29]). *R_f_* 0.24 (C). ^1^H NMR (D_2_O) δ: 3.47–3.35 (m, 1H, CH(CH_3_)); 3.22–3.07 (m, 6H, NH_2_CH_2_ + CH_2_NHCH_2_); 2.17–2.03 (m, 2H, NH_2_CH_2_CH_2_); 1.86–1.60 (m, 4H, CHCH_2_CH_2_); 1.31 (d, 3H, *J* = 6.6 Hz, CH_3_). ^13^C NMR (D_2_O) δ: 47.91, 47.84, 45.18, 37.22, 31.47, 24.37, 22.50, 17.94. Found, %: C 35.70; Н 9.18; N 15.63. C_8_H_24_Cl_3_N_3_. Calculated, %: C 35.77; Н 9.00; N 15.64. HRESIMS: calculated for C_8_H_22_N_3_ [M + H]^+^: *m/z* 160.1808. Found: *m/z* 160.1806.

### 4.3. Synthesis of 2,9-Diamino-4-azadecane (1,8-Me_2_Spd)

*N^2^-(Benzyloxycarbonyl)-N^9^-(tert-butyloxycarbonyl)-2,9-diamino-5-azadecane* **(16)**. A mixture of **15** (2.9 g, 7.2 mmol), *N*-(benzyloxycarbonyl)-3-amino-1-bromobutane (2.31 g, 8 mmol), and K_2_CO_3_ (2.34 g, 17 mmol) was stirred in DMF (25 mL) for 16 h at 50 °C followed by the addition of PhSH (1.5 mL, 14.7 mmol) and K_2_CO_3_ (2.34 g, 14.5 mmol). After stirring for an additional 12 h at 20 °C, the salts were filtered off and washed with DMF, and combined DMF filtrates were concentrated in vacuo. The residue was treated with a mixture of EtOAc (25 mL) and H_2_O (15 mL), the water phase was extracted with EtOAc (3 × 5 mL), and combined EtOAc extracts were washed with H_2_O (5 mL), brine (10 mL), and dried (MgSO_4_). The solvent was distilled off in vacuo and the residue was purified on a SiO_2_ column eluting subsequently with CHCl_3_, CHCl_3_–MeOH, 100:1, and then CHCl_3_–MeOH, 95:5. Fractions containing **15** were evaporated to dryness in vacuo to afford a self-solidified oil, which was treated with a hexane/ether mixture (2/1, 15 mL) and left for 4 h at +4 °C. The resultant solid was filtered off, washed with hexane, and dried in the air to give **15** (1.67 g, 57%) as the colorless crystals. An analytical sample was recrystallized from Et_2_O/hexane, mp 55–55.5 °C, *R_f_* 0.47 (D). ^1^H NMR (CDCl_3_) δ: 7.38–7.29 (5H, m, C_6_H_5_); 5.49 (1H, bs, CbzNH); 5.07 (2H, s, CH_2_C_6_H_5_); 4.47 (1H, bs, NHBoc); 3.87–3.73 (1H, m, CbzNHCH); 3.67–3.53 (1H, m, CHNHBoc); 2.72–2.51 (4H, m, CH_2_NH_2_CH_2_); 1.72–1.62 (m, 1H, ½ CH_2_CHNHCbz), 1.58–1.35 (14H, m, ½ CH_2_CHNHCbz + CH_2_CH_2_NHBoc + C(CH_3_)_3_); 1.17 (3H, d, *J* = 6.0 Hz, CbzNHCH(CH_3_)); 1.05 (3H, d, *J* = 6.0 Hz, CH(CH_3_)NHBoc). ^13^C NMR (CDCl_3_): δ 216.31, 156.38, 155.84, 137.23, 128.88, 128.43, 128.40, 79.27, 71.95, 66.78, 50.09, 46.78, 46.37, 37.01, 35.34, 28.86, 26.89, 21.61. Calculated, %: C 64.84; H 9.15; N 10.31. C_22_H_37_N_3_O_4_. Found, %: C 64.68; H 9.17; N 10.21.

*N^2^-(Benzyloxycarbonyl)-2,9-diamino-5-azadecane dihydrochloride* **(17)**. To a cooled (0 °C) solution of **10** (1.6 g, 3.9 mmol) in MeOH (12 mL), HCl/MeOH (9 M, 2.8 mL) was added, and the mixture was kept for 1 h at 20 °C followed by evaporation to dryness in vacuo. The residue was dissolved in EtOH (3 mL) and precipitated with an excess of Et_2_O that, after drying in vacuo over P_2_O_5_/KOH, gave **11** (1.16 g, 78%). An analytical sample was recrystallized from dry EtOH, mp 157–159 °C, *R_f_* 0.19 (B). ^1^H NMR (D_2_O): δ 7.39–7.30 (5H, m, C_6_H_5_); 5.03 (2H, s, PhCH_2_); 3.66–3.58 (1H, m, CbzNHCH(CH_3_)); 3.33–3.24 (1H, m, CHNH_2_); 2.97–2.79 (4H, m, CH_2_NHCH_2_); 1.83–1.72 (1H, m, 1/2 CbzNHCHCH_2_); 1.70–1.55 (4H, m, CH_2_CH_2_CHNH_2_); 1.54–1.47 (1H, m, 1/2 CH_2_CHNHCbz); 1.21 (3H, d, *J* = 6.0 Hz, CbzNHCH(CH_3_)); 1.11 (3H, d, *J* = 6.1 Hz, CH(CH_3_)NH_2_). ^13^C NMR (D_2_O): δ 158.45, 137.10, 129.27, 128.81, 127.95, 67.36, 47.68, 47.46, 45.28, 44.96, 33.13, 31.27, 22.25, 20.32, 17.71. Calculated, %: C 53.68; H 8.22; N 11.05. C_17_H_31_Cl_2_N_3_O_2_. Found, %: C 53.59; H 8.31; N 10.97.

*2,9-Diamino-5-azadecane trihydrochloride, 1,8-Me_2_Spd* **(6)**. Dihydrochloride **11** (0.8 g, 2.1 mmol) was mixed with 2 М NaOH (5 mL) and extracted with CH_2_Cl_2_ (3 × 5 mL). Combined organic extracts were washed with 1 М NaHCO_3_ (5 mL), H_2_O (3 mL), brine (5 mL), dried (K_2_CO_3_), filtered, and concentrated in vacuo, and the residue was dried in vacuo over P_2_O_5_ to give an *N*^2^-(benzyloxycarbonyl)-2,9-diamino*-*5-azadecane free base as a viscous oil. Pd-black in MeOH (~0.5 mL) was added to a solution of the above *N*-Cbz-triamine in a mixture of AcOH–MeOH (1:1, 6 mL total), and hydrogenation was carried out at atmospheric pressure. The catalyst was filtered off and washed with MeOH, and the combined filtrates were concentrated in vacuo. The residue was dissolved in EtOH and, upon the addition of HCl/EtOH (7 M, 0.8 mL), was evaporated to dryness in vacuo, and the residue was dried in vacuo over KOH. Recrystallization from EtOH afforded **6** (0.69 g, 87%) as the colorless crystals, mp 252–253°C (soften at 194–196 °C), *R_f_* 0.29 (C). ^1^H NMR (D_2_O) δ: 3.56–3.34 (m, 2H, 2 × CH(CH_3_)); 3.24–3.06 (m, 4H, CH_2_NHCH_2_); 2.20–1.90 (m, 2H, ½ NH_2_CHCH_2_ + ½ NH_2_CHCH_2_CH_2_NH); 1.87–1.58 (m, 4H, ½ NH_2_CHCH_2_ + ½ NH_2_CHCH_2_CH_2_NH + NHCH_2_CH_2_CH_2_); 1.34 (d, 3H, *J* = 6.6 Hz, CH_3_), 1.31 (d, 3H, *J* 6.6 Hz, CH_3_). ^13^C NMR (D_2_O) δ: 47.91, 47.85, 45.96, 44.58, 31.48, 30.06, 22.53, 17.93, 17.89. Found, %: C 36.95; Н 9.35; N 14.31. C_9_H_26_Cl_3_N_3_ × 0.5H_2_O. Calculated, %: C 37.05; Н 9.32; N 14.41. HRESIMS: calculated for C_9_H_24_N_3_ [M + H]^+^: *m/z* 174.1665. Found: *m/z* 174.1970.

### 4.4. Plasmid Construction

The plasmid pET-15b-2c-His-mAZ (Figure S1) encoding an *N*-terminally His-tagged OAZ1 was constructed using a two-step procedure. First, an additional cistron, like the one described in [60], was introduced into a pET-15b vector by the excision of a small fragment between *Xba*I and *Nco*I sites and the ligation of a preannealed duplex of synthesized prephosphorylated oligonucleotides (5′-CTAGAGGGTATTAATAATGTATCGATTAAATAAGGAG-GAATAAAC-3′) and (5′-CATGGTTTATTCCTCCTTATTTAATCGATACATTATTA-ATACCCT-3′) to give the pET-15b-2c vector. Then, the mOAZ1-encoding region from the plasmid pQE30-mAZ [58] (a kind gift of Dr. T.A. Keinänen, University of Eastern Finland, Kuopio, and Prof. O. Jänne, University of Helsinki, Finland) was amplified using primers (ATTACTCGAGGTGAAATCCTCCCTGCA) and (ATTACTCGAGTTAGTCCTCCTC-ACCCGGGT) and cloned into the *Xho*I site of the pET-15b-2c vector. The structure of all constructed plasmids was confirmed by sequencing.

### 4.5. The Expression and Purification of mOAZ1 Protein

Rosetta (DE3) *Escherichia coli* strain (Novagen) was transformed with the plasmid pET-15b-2c-His-mAZ encoding *N*-terminally His-tagged protein. The cells bearing the target plasmid were grown in 5 mL of a Lisogeny broth (LB) medium supplemented with 150 mg/L ampicillin (A150) and 15 mg/L chloramphenicol (C15) at 37 °C overnight. An aliquot of 2 mL was harvested by centrifugation (3200× *g*, 10 min), the pellet was resuspended in 500 mL of a fresh LB medium supplemented with A150 and C15, and the cells were grown at 37 °C. When optical density at 550 nm reached 0.5, isopropyl-β-D-thiogalactopyranoside was added to a final concentration of 1 mM, and the cells were grown for an additional 3 h and harvested by centrifugation (3200× *g*, 15 min) at 4°C. The cell pellet was resuspended on ice in 20 mL of buffer A (25 mM Tris–HCl, pH 7.5, 350 mM NaCl, 10% (*v/v*) glycerol, and 1 mM 2-mercaptoethanol) supplemented with 0.5% (*v/v*) Triton X-100, and protease inhibitors phenylmethylsulfonyl fluoride (PMSF, 1 mM) and a protease inhibitor cocktail (P8849, Sigma, St. Louis, MO, USA). The lysate was sonicated on ice by ten 46 s impulses with 90 s intervals. After removal of cell debris by centrifugation (8200× *g*, 15 min) at 4 °C, the clarified lysate was applied onto a 2 mL column with Ni-NTA-agarose (Novagen) pre-equilibrated with buffer A. The resin was washed with buffer A supplemented with the protease inhibitors and increasing concentrations of imidazole (10, 30, and 50 mM) (15 mL each), and the protein was eluted with the same buffer with 200 mM imidazole; 1 mL fractions were collected, and the protein was quantitated using a Bradford reagent. The fractions containing the highest levels of the protein were pooled and dialyzed against buffer B (25 mM potassium phosphate, рН 7.5, 300 mM КCl, 5% (*v/v*) glycerol, 1 mM 2-mercaptoethanol, and 1 mM PMSF) for 4 h and then against buffer C (25 mM potassium phosphate, рН 7.5, 150 mM КCl, 50% (*v/v*) glycerol, 1 mM 2-mercaptoethanol, and 1 mM PMSF) overnight. The yield of the *N*-terminally tagged antizyme was 4 mg per 1 L of the culture.

When required, the *N*-terminal His-tag was removed using a Novagen Thrombin Cleavage Capture kit, according to the manufacturer’s protocol. Briefly, 200 μg of the protein was incubated with thrombin overnight at 4 °C, the reaction mixture was applied onto a Ni-NTA-agarose column (0.5 mL), and the flowthrough was dialyzed as described above. The purified 6xHis-mOAZ1 eluted from the Ni-NTA-agarose column was practically homogeneous, as shown by SDS-PAGE electrophoresis (Figure S1).

### 4.6. Isothermal Titration Calorimetry (ITC)

For the reproducible isothermal titration calorimetry (ITC) experiments, only freshly purified samples of mOAZ1 turned out to be suitable. The thermodynamic parameters of Spm binding to mOAZ1-6xHis, mOAZ1, and 6xHis-mOAZ1 were measured using a MicroCal iTC200 instrument (GE Healthcare), as described elsewhere [61]. Experiments were carried out at 31 °C in a buffer containing 25 mM potassium phosphate, рН 7.5, 150 mM KCl, 25% (*v*/*v*) glycerol, 1 mM 2-mercaptoethanol, and 1 mM PMSF. Aliquots (2.5 μL) of Spm solution were injected into a 0.2 mL cell containing the protein solution to achieve a complete binding isotherm. Protein and Spm concentrations were 20 μM and 200 μM, respectively. The heat of dilution was measured by injecting the Spm solution into the buffer solution or by additional injections of the Spm solution after saturation; the values obtained were subtracted from the heat of the reaction to obtain the effective heat of the binding. The resulting titration curves were fitted using MicroCal Origin software, assuming a single binding site. Affinity constants (*K*_a_) and enthalpy variations (Δ*H*) were determined, and the entropy variations (Δ*S*) were calculated by the equation *T*Δ*S* = Δ*H* + *RT*ln*K*_a_.

### 4.7. The Stem-Loop Region of mOAZ1 mRNA Melting Point Experiments

UV thermal denaturation data were obtained using a Hitachi U-2900 spectrophotometer equipped with a Peltier temperature controller. The concentration of model 72-mer-2’-O-Me-oligoribonucleotide L-OM, containing the +1-frameshifting site, hairpin, and pseudoknot of the mOAZ1 mRNA, was 1 µM. Samples were dissolved in a 50 mM Tris-HCl pH 7.5, 50 mM NaCl buffer. The concentration of oligonucleotide was determined spectrophotometrically at λ = 260 nm. Samples were heated to 90 °C for 10 min, cooled slowly to room temperature, and stored at 5 °C for at least 18 h before the measurements were performed. Denaturation curves were acquired at 260 nm for the duplexes at a rate of 0.5°C*/*min, within a range of 30–90 °C. The *T*_m_ values were determined from the polynomial fitting of the observed curves and taken as the temperatures corresponding to the half-dissociation of the complex [62]. The first derivative of absorption with respect to the temperature and dA/dT of the melting curve was computer-generated by GraphPad Prism 7.0 software and used to determine *T*_m_.

## Figures and Tables

**Table 1:** Thermodynamic parameters of Spm binding with mOAZ1 and mOAZ1 carrying either a *C*- or *N*-terminal His-tag, determined by isothermal titration calorimetry ^a^.

Stoichiometry mOAZ1/Spm	*K_a_*^b^, M^−1^	*K_d_*^c^, µM	Δ*H* ^d^, kcal/mole	TΔS ^e^, kcal/mole
mOAZ1‒6XHis-tag *				
2:1	9.2 × 10^5^	1.1	−3.7	4.5
6XHis-tag‒mOAZ1				
No binding ^f^				
mOAZ1				
2:1	2.7 × 10^6^	0.37	−3.6	5.5

^a^ All measurements were performed three to five times in a buffer containing 25 mM potassium phosphate buffer, рН 7.5, 150 mM KCl, 25% (*v*/*v*) glycerol, 1 mM 2-mercaptoethanol, and 1 mM PMSF. ^b^ *K_a_*—affinity constant; standard deviation did not exceed ±25%. ^c^ *K_d_*—dissociation constant; calculated as 1/*K_a_*. ^d^ Δ*H*—enthalpy change; standard deviation did not exceed ±20%. ^e^ *T*Δ*S*—entropy change; calculated from the equation *T*Δ*S =* Δ*H + RT*ln*K_a_*. ^f^ No binding means that under the experimental conditions, it was impossible to determine *K_d_*. * Data from [31].

**Table 2:** The effect of Spd and its *C*-methylated analogs on the melting temperature (*T*_m_) of 72-mer 2′-O-Me-oligoribonucleotide (L-OM), containing a +1 frameshifting site, hairpin, and pseudoknot of the mOAZ1 mRNA. *T*_m_ measurements were performed in at least three separate experiments, with 1 μM L-OM (5′-UGG UGC UCC UGA UGU CCC UCA CCC ACC CCU GAА GAU CCC AGG UGG GCG AGG GAA CAG UCA GCG GGA UCA CAG-3′) in 50 mM Tris-HCl pH 7.5, 50 mM NaCl buffer.

Polyamine Concentration, μM	*T*_m_, °C					
	Spd ^a^	1-MeSpd ^a^	2-MeSpd ^a^	3-MeSpd ^a^	8-MeSpd	2,2-Me_2_Spd
0	65.5 ± 0.6					
20	73.7 ± 0.6	73.0 ± 0.4	70.5 ± 0.5 ***	71.9 ± 0.5 *	72.7 ± 0.4	71.6 ± 0.9
50	75.3 ± 0.4	74.0 ± 0.3	73.4 ± 0.4 *	75.2 ± 0.4	76.2 ± 0.7	74.4 ± 0.6
200	79.7 ± 1.1	80.2 ± 0.6	77.5 ± 0.5 **	79.4 ± 0.5	81.3 ± 1.4	76.7 ± 0.9

Results are means ± SD, *n* = 3. *, **, and *** refer to a statistical significance of *p* < 0.05, *p* < 0.01, and *p* < 0.001, respectively, as compared to the Spd group of the same concentration. ^a^ data from [31].

## Data Availability

All data are available in the main text or the Supplementary Materials.

## Supplementary Materials

The following supporting information can be downloaded at https://www.mdpi.com/article/10.3390/biom13060916/s1, Figure S1: The purified *N*-terminally His-tagged mOAZ1 protein used in the study; Figure S2: A schematic representation of His-OAZ1 expression vector pET-15b-2c-His-mAZ; Figure S3: Interaction of mOAZ1-His_6_, His_6_-mOAZ1 and mOAZ1 with Spm measured by ITC; Figures S4–S13: ^1^H- and ^13^C-NMR spectra of 2-MeSpd, 2,2-Me_2_Spd, 3-MeSpd, 8-MeSpd, and 1,8-Me_2_Spd.
