# Supplementary figures and images for: C-Methylated Spermidine Derivatives: Convenient Syntheses and Antizyme-Related Effects
Source: Biomolecules. 2023 May 31;13(6):916. doi: 10.3390/biom13060916 (PMC10296195; doi:10.3390/biom13060916)

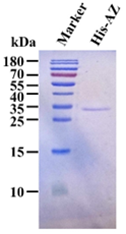

Supplement: Supplementary file 1 [file biomolecules-13-00916-s001.zip › Figure S1.tif]

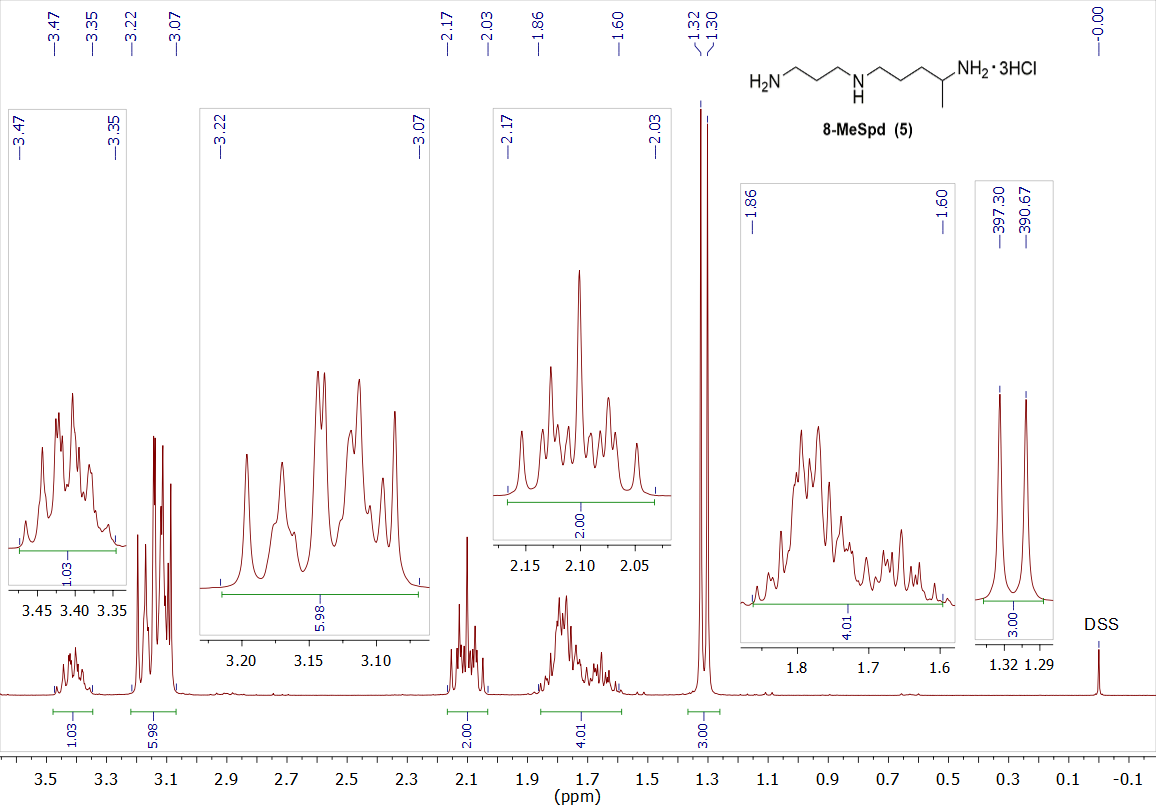

Supplement: Supplementary file 1 [file biomolecules-13-00916-s001.zip › Figure S10.tiff]

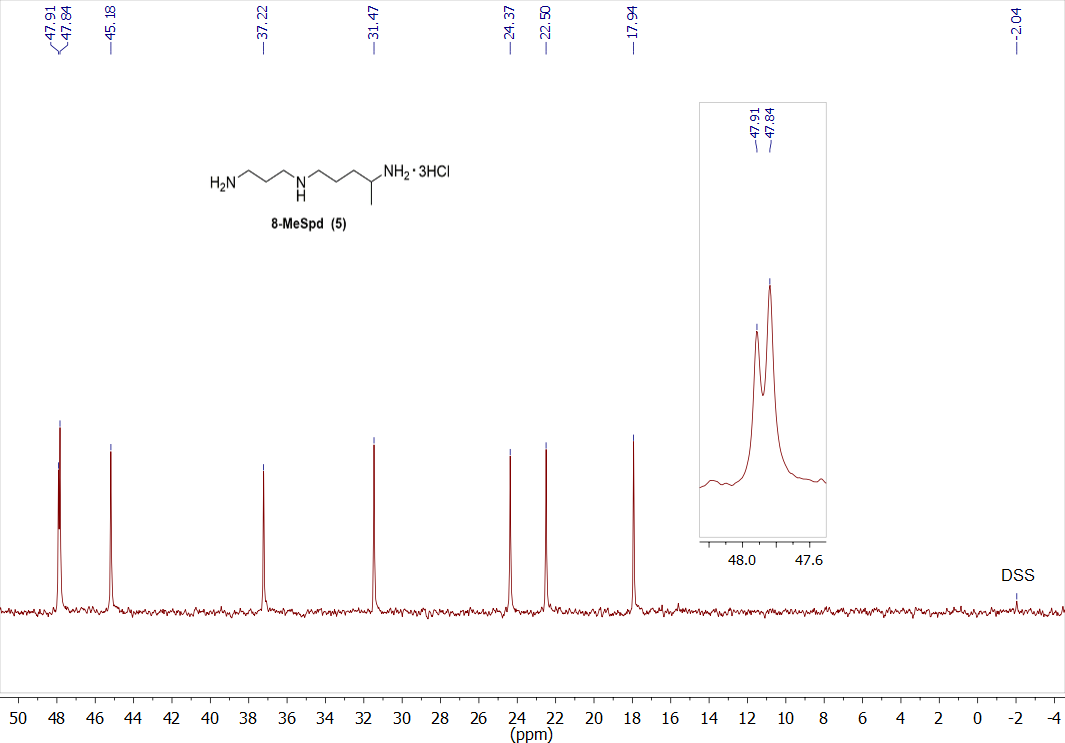

Supplement: Supplementary file 1 [file biomolecules-13-00916-s001.zip › Figure S11.tiff]

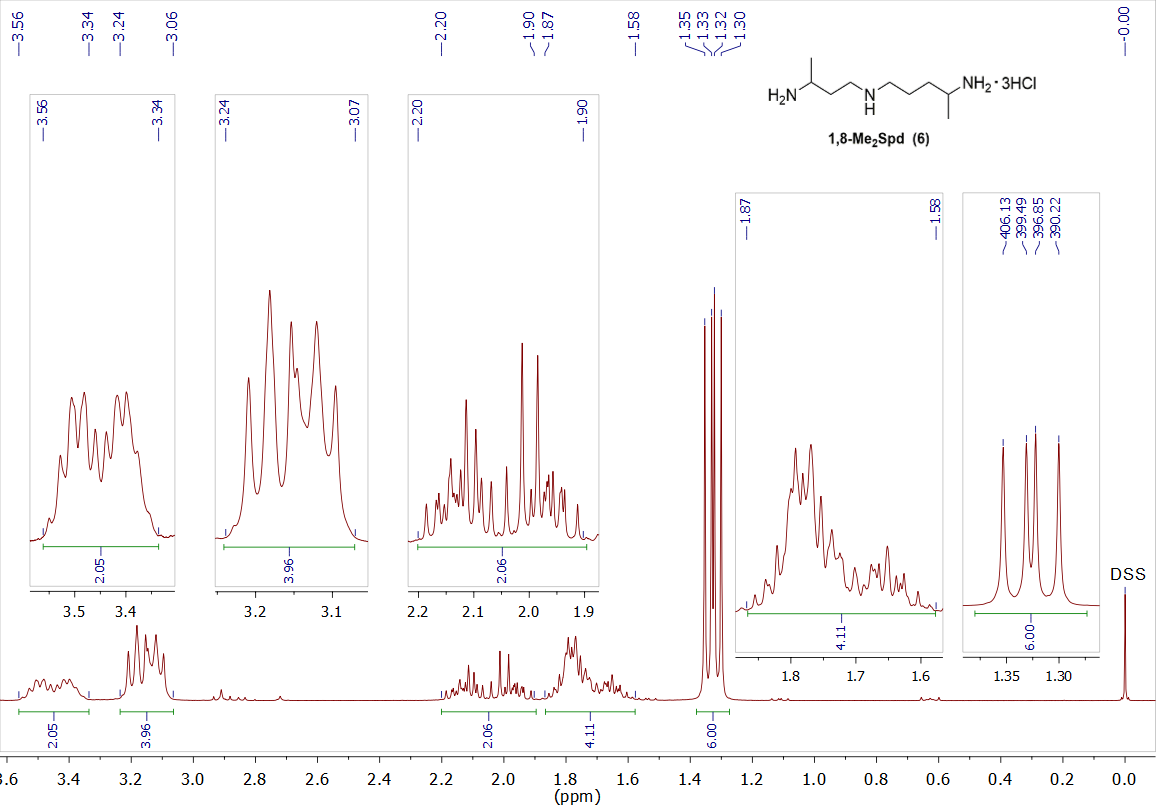

Supplement: Supplementary file 1 [file biomolecules-13-00916-s001.zip › Figure S12.tiff]

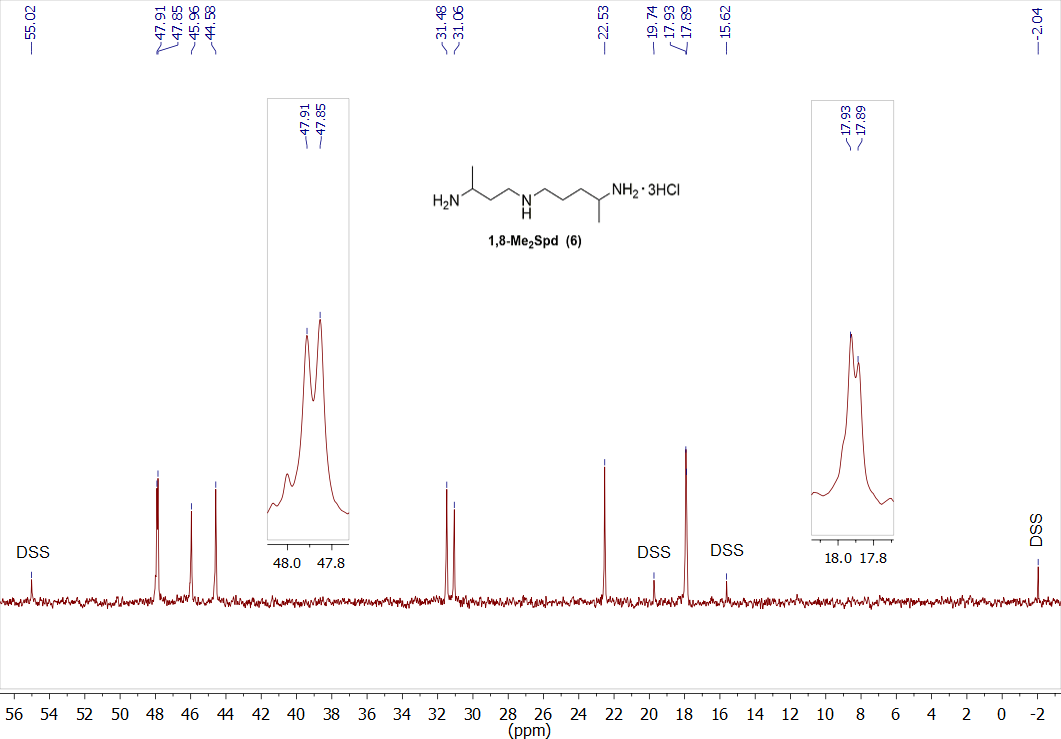

Supplement: Supplementary file 1 [file biomolecules-13-00916-s001.zip › Figure S13.tiff]

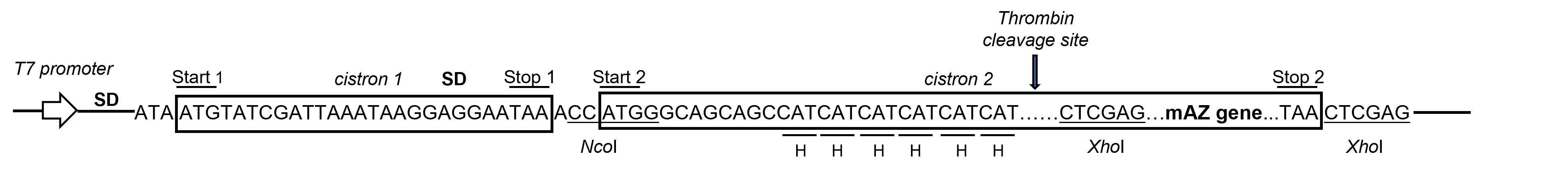

Supplement: Supplementary file 1 [file biomolecules-13-00916-s001.zip › Figure S2.tif]

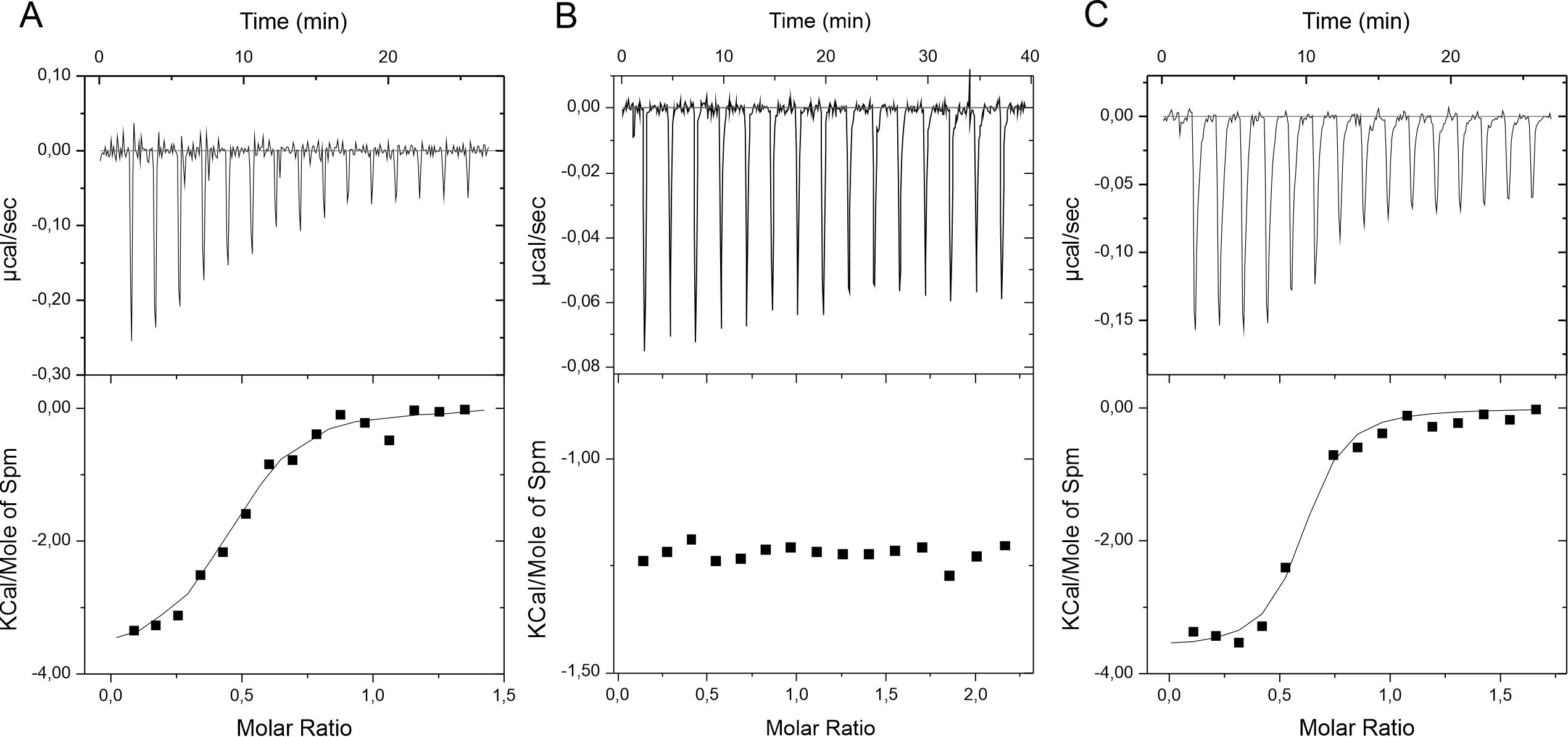

Supplement: Supplementary file 1 [file biomolecules-13-00916-s001.zip › Figure S3.tif]

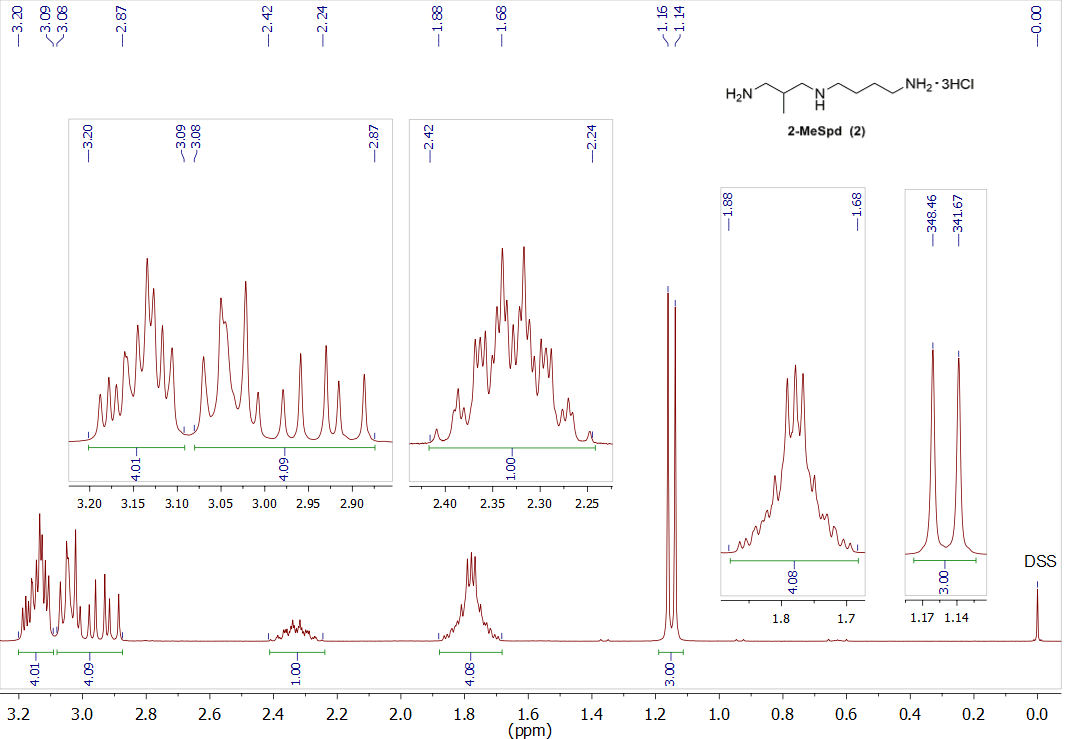

Supplement: Supplementary file 1 [file biomolecules-13-00916-s001.zip › Figure S4.tiff]

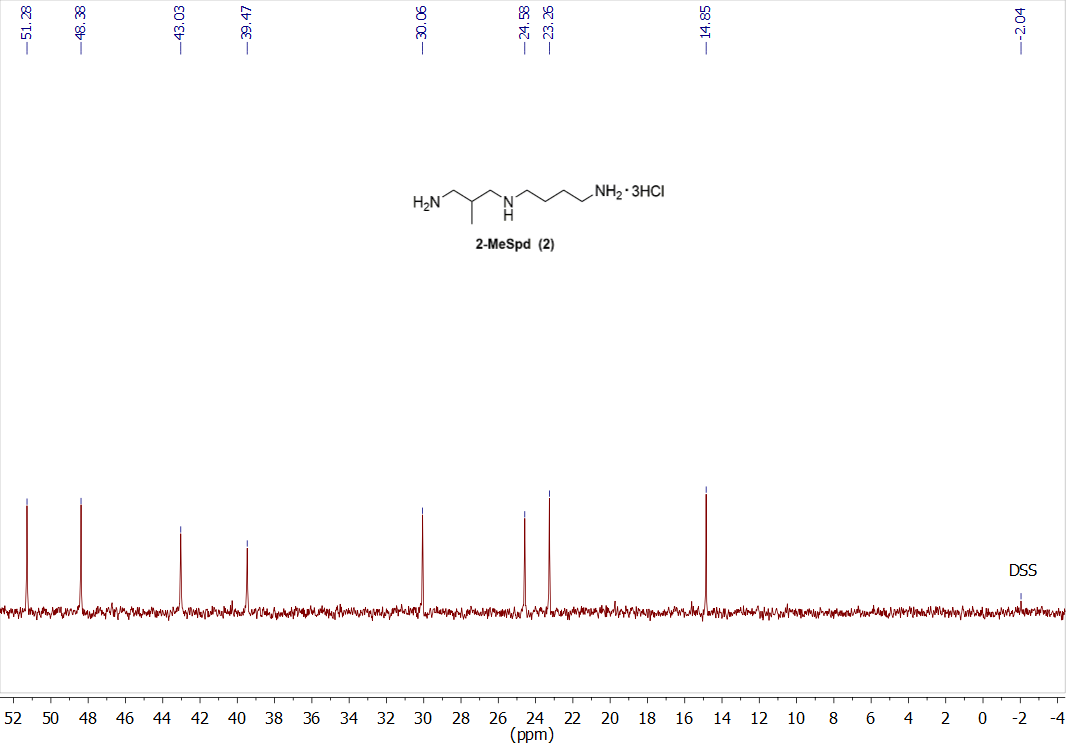

Supplement: Supplementary file 1 [file biomolecules-13-00916-s001.zip › Figure S5.tiff]

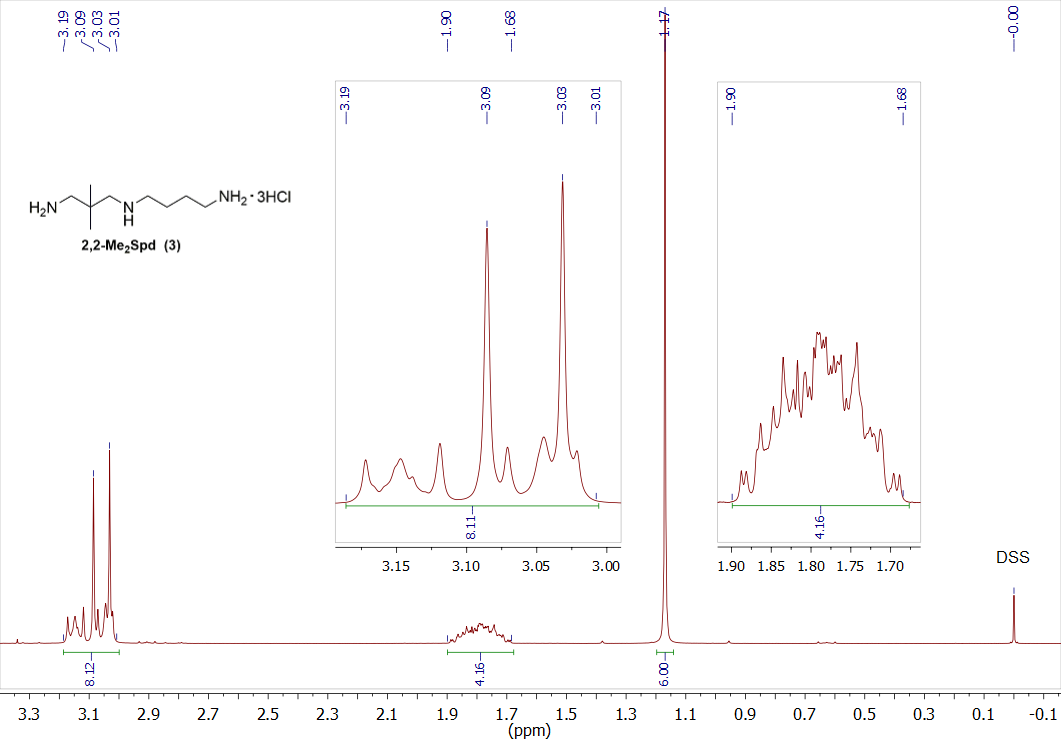

Supplement: Supplementary file 1 [file biomolecules-13-00916-s001.zip › Figure S6.tiff]

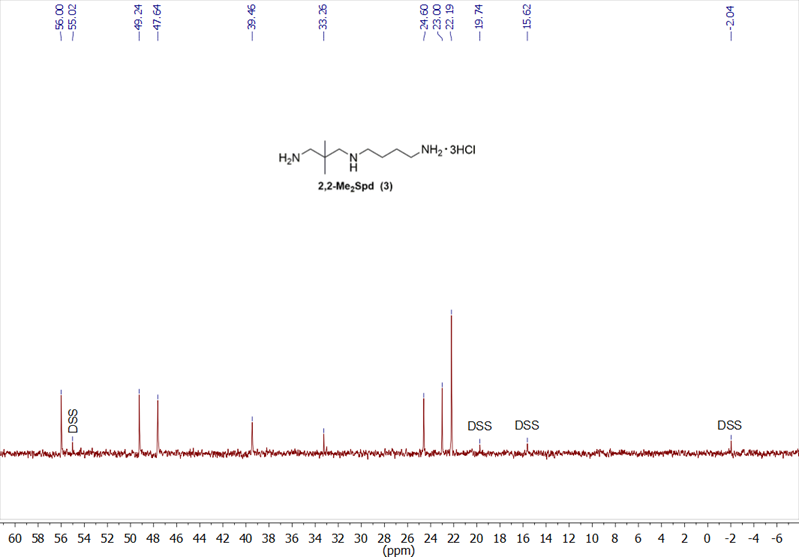

Supplement: Supplementary file 1 [file biomolecules-13-00916-s001.zip › Figure S7.tif]

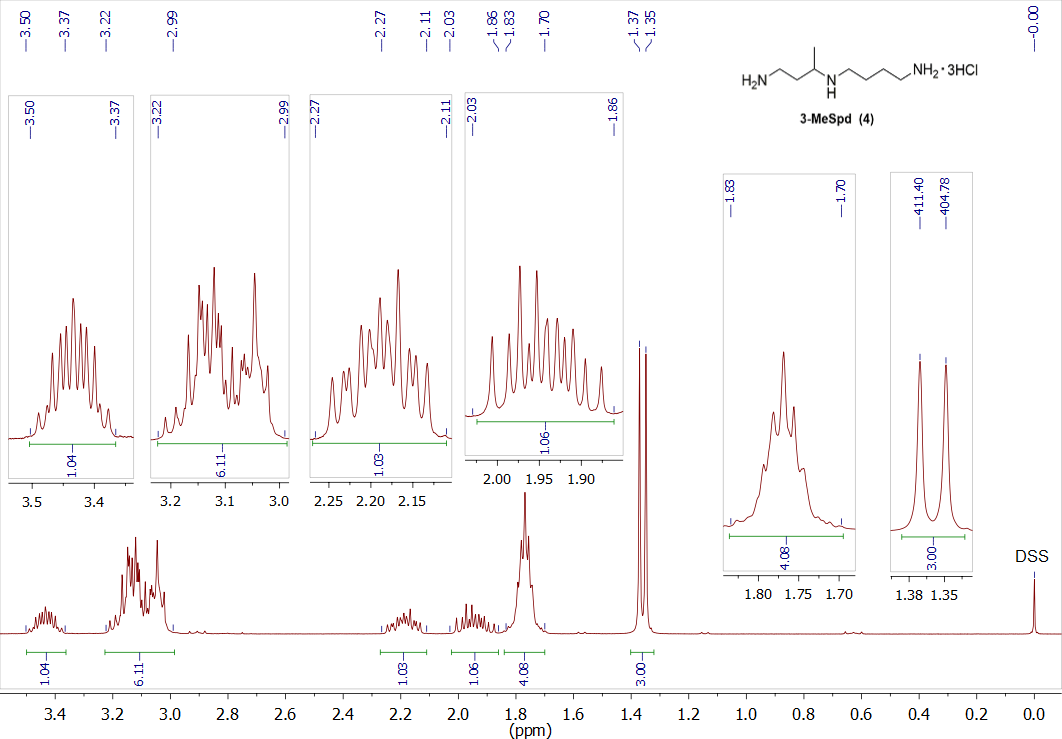

Supplement: Supplementary file 1 [file biomolecules-13-00916-s001.zip › Figure S8.tiff]

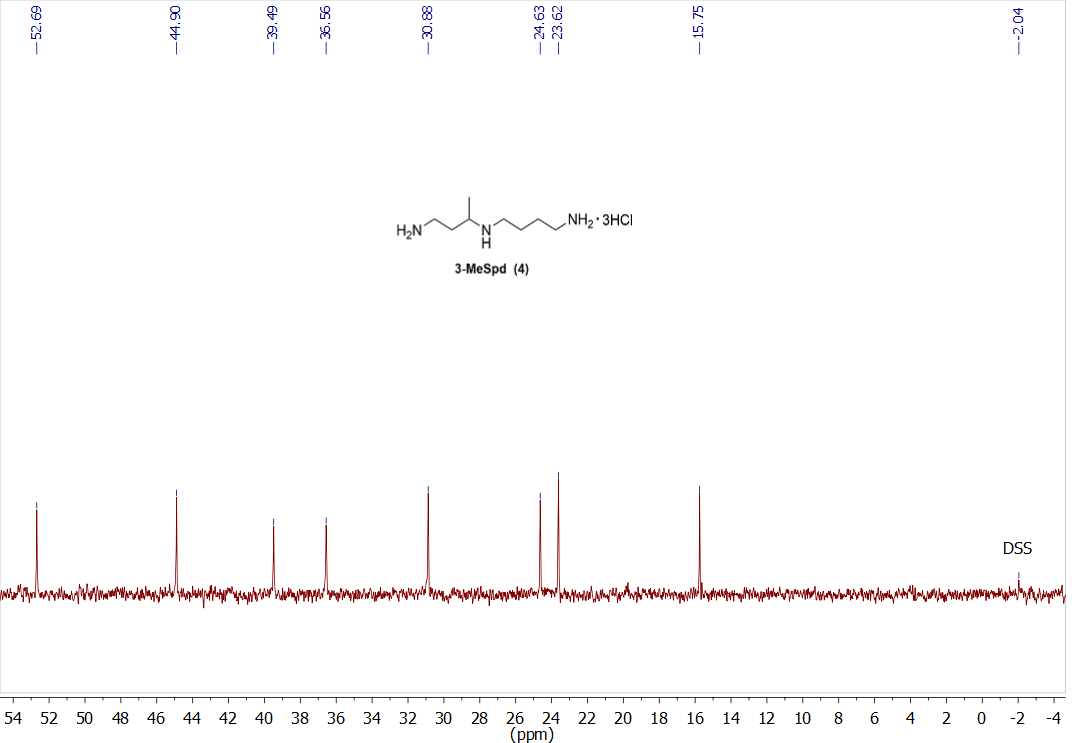

Supplement: Supplementary file 1 [file biomolecules-13-00916-s001.zip › Figure S9.tiff]
